# Optimized real-time fluorescence PCR assay for the detection of porcine Circovirus type 3 (PCV3)

**DOI:** 10.1186/s12917-020-02435-y

**Published:** 2020-07-17

**Authors:** Lin Yuan, Yingyi Liu, Yana Chen, Xiaoxue Gu, Hao Dong, Shuo Zhang, Tao Han, Zhi Zhou, Xiaohui Song, Chuanbin Wang

**Affiliations:** China Animal Disease Control Center, OIE Porcine Reproductive and Respiratory Syndrome Reference Laboratory, No. 17 Tiangui Street, Biomedical Base, Daxing District, Beijing, 102618 China

**Keywords:** Porcine circovirus type 3, Real-time PCR, Virus

## Abstract

**Background:**

Porcine circovirus type 3 (PCV3) has been an emerging porcine virus spread around the world. The conserved DNA sequence of PCV3 enabled good performance in molecular biological assays.

**Result:**

In this study, we developed a real-time fluorescence PCR assay for the detection of PCV3. The conserved region within Capsid genome of PCV3 was selected for the design of primer pairs and probes. After optimizing, a primer pair and probe was screened, providing high sensitivity (10 copies/μL) and specificity (no cross reaction with other porcine viruses or common bacterium). In addition, this method was applied in the detection of 110 clinical samples, and the performance was compared with other previously reported PCR and real-time PCR methods. This method provided higher detection rate.

**Conclusion:**

A real-time fluorescence PCR assay has been developed for the detection of PCV3, with high sensitivity and specificity, exhibiting good performance in detecting clinical samples.

## Background

Porcine circovirus type 3 (PCV3) has been an emerging porcine virus since 2016 [1, 2]. This virus has been widely spread in the worldwide, including USA, Europe and Korea [3, 4]. PCV3 has been identified as one of the pathogens for Porcine Circovirus Associated Disease (PCVAD), presenting as cardiac and multisystemic inflammation, porcine dermatitis and nephropathy syndrome (PDNS), reproductive failure and aborted fetuses [1]. Although the mortality of PCVAD has been low, this disease would lead to large economic loss to porcine industry. Recently, the epidemiology of PCV3 has been also reported in several regions of China [5, 6].

PCV3 is a single-stranded circular DNA virus. The genome of PCV3 consists of approximately 2000 bp nucleotides, with two inversely arranged ORFs encoding replicase (296 aa) and capsid (214 aa) proteins, respectively [2]. The sequence encoding capsid has been conserved, which was naturally applied as the target for molecular biological diagnosis [1, 7]. Several previous studies have reported the PCR and real-time PCR methods targeted the sequence of capsid.

Here, we have developed a TaqMan-based real-time PCR assay for the detection of PCV3. This method presented good sensitivity and specificity. In addition, the performance of this method for the detection of clinical samples has been compared with other previously reported methods. A good agreement was achieved, with even higher detection rate.

## Results

### The development of real-time PCR assay

The optimal concentration of primers and probe giving the best performance was determined as 0.6 μmol/L forward and reverse primers (PCV3-F and PCV3-R, Table 1), and 0.25 μmol/L probe (PCV3-P). In the final reaction system of 20 μL, 1.2 μL of primers pair (10 μmol/L) and 0.5 μL of probe (10 μmol/L) was added to obtain the optimal concentration.

**Table 1:** The primer and probe sequences

Methods	Primer and probe sequences		Length	Readout
PCR	F:5′-CCA CAG AAG GCG CTA TGT C-3′ R: 5′-CCG CAT AAG GGT CGT CTT G-3′	Palinski, 2016 [1]	330 bp	Electrophoresis
qPCR	F: 5′-CGCATAAGGGTCGTCTTGGA-3′ R: 5′-CMGCTCAGCAAACAAAAACTATGTTC-3′ P: 5′-FAM-TCCAGGCGCCGTCTAGATCTATGGC-BHQ1–3′	This study	95 bp	Real-time fluorescence
qPCR	F: 5′-CGGACTTGTAACGAATCCAAACT-3′ R: 5′-GGAGCATTTATGCCCCGGAAA-3′ P: FAM-5′-CTTTSGTGCCGTAGAAGTCTGTCATTCCA-3′-Eclipse	Wang, 2017 [7]	78 bp	Real-time fluorescence

*F* forward primer, *R* reserve primer, *P* probe

### Sensitivity

**Fig. 1:**
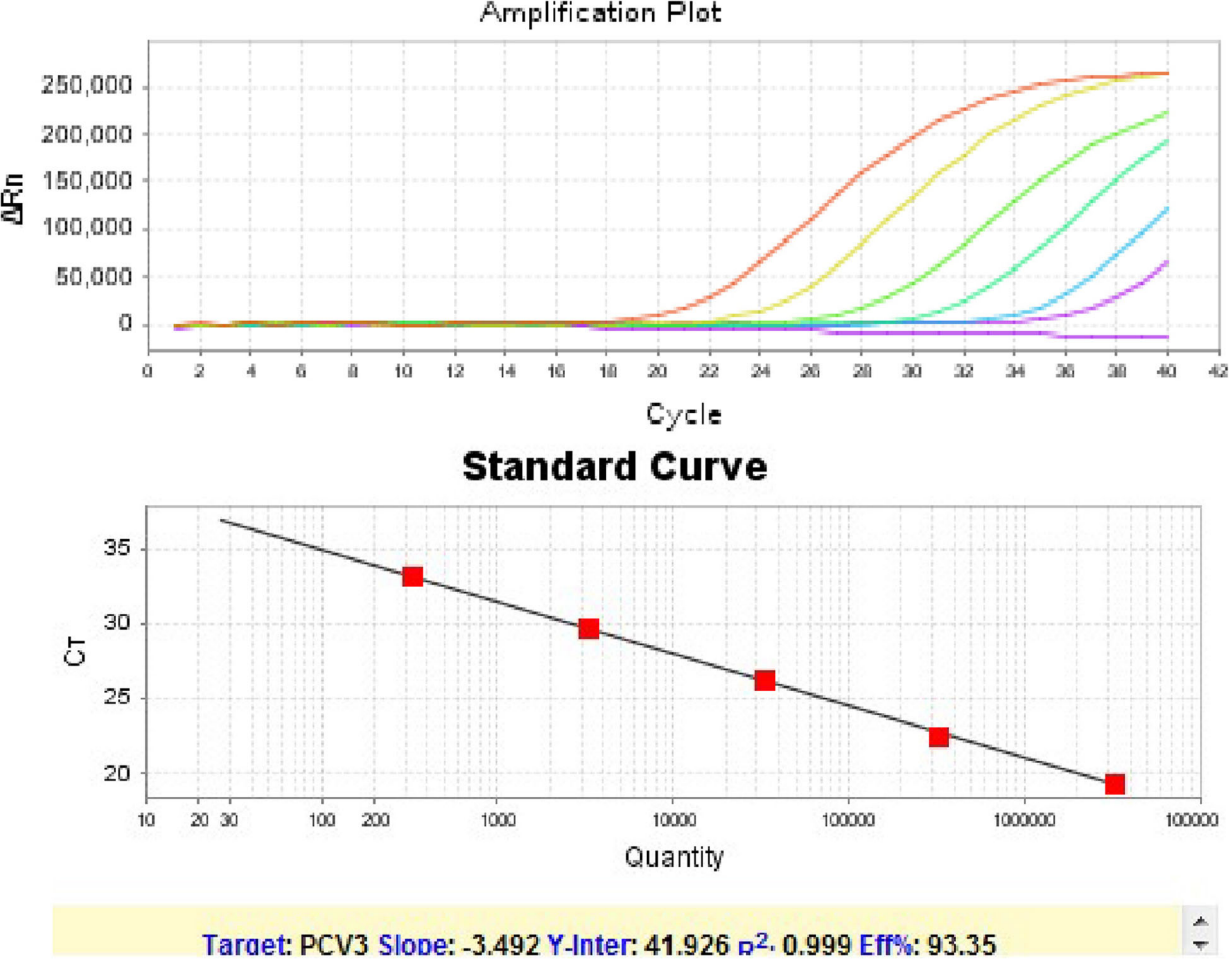
The amplification plot of PCV3 capsid plasmid with the concentration of 10^6^, 10^5^, 10^4^, 10^3^, 10^2^ and 10^1^ copies/μL. The standard curve plotted against the Ct values in amplification plot (linear range: 10^2^–10^6^ copies/μL, R^2^ = 0.999)

The sensitivity of this method was determined with ten-fold diluted plasmid solutions, and the plasmid concentration was ranged from 10^6^ to 10^1^ copies/μL (Fig. 1). Typical amplification curve could be obtained. The linear coefficent curve could be plotted over a 10^5^ dilution range (10^2^–10^6^ copies/μL), with R^2^ value of 0.999 and reaction efficiency of 93.35%. The sensitivity could be as low as 10 copies/μL. The sensitivity test was repeated in triplicate. The Ct value was provided and the mean ± standard deviation (SD) was calculated. The SD of the triplicate tests was relatively small, indicating good repeatability (Supporting information: Figure S1 and Table S1).

### Specificity

**Fig. 2:**
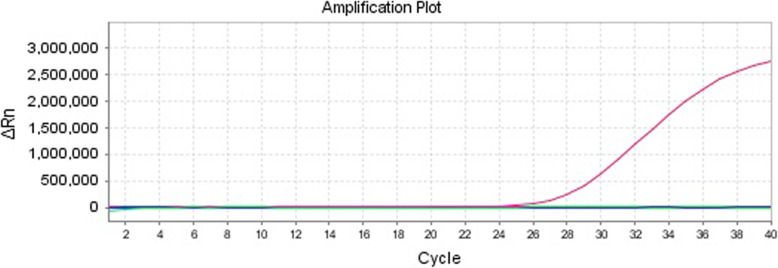
The specificity of real-time fluorescence PCV3 PCR system. This assay showed no cross-reaction with other swine viruses and bacterium, including PCV2, PRV, PRRSV-EU, PRRSV-NA, CSFV, PPV, *E. coli*. and *App.*

Several common swine viruses were collected and detected for verifying the specificity of this method. The included viruses were PCV2, PRV, PRRSV-EU, PRRSV-NA, CSFV, PPV, *E.coli.* and *Actinobacillus pleuropneumoniae (App.)*. The negative control was SPF porcine serum and the positive control was PCV3 capsid plasmid. According to the results (Fig. 2), this method showed a good specificity without cross-reaction with other viruses and bacterium.

### Clinical samples and comparison with other methods

A total of 110 tissue samples were collected from diseased pigs with dermatitis, respiratory and reproductive symptoms. The diseased pigs were from both pig breeding farm and slaughter houses in nationwide. The detection results of three methods were provided (Table 2). In the common PCR method (Palinski, 2016), the results were determined with electrophoresis, and there were 7 positive samples and 103 negative samples. In Wang’s method, there were 20 positive samples (Ct value ranged from 26.71–36.74) and 90 negative samples. In our method, there were 28 positive samples (Ct value ranged from 26.43–36.98) and 82 negative samples. The interpretation was made based on both Ct value and the shape of amplification curve. Two real-time PCR methods were compared. A relatively high coincidence rate of 92.73% and moderate Kappa value of 0.79 was obtained (Table 2).

**Table 2:** The comparison results for clinical samples detection

Test	qPCR (This study)		Agreement (%)	Kappa
	Positive	Negative		
qPCR (Wang)				
Positive	20	0		
Negative	8	82	92.73	0.79
PCR (Palinski)				
Positive	7	0		
Negative	21	82	80.91	0.33

## Discussion

PCV3 has been a conserved DNA virus and all the existing PCV3 virus strains shared a homology of higher than 90%. Therefore, a good performance could be obtained with the molecular biological assay. In previous studies, many fluorescence PCR amplification methods have been reported. We have also compared these methods with ours. These methods all presented high sensitivity (about 10 copies/μL) and good specificity (no cross-reaction with other porcine viruses) [1, 7].

In veterinary field, the samples were always complex, such as pooled tissues. Therefore, the availability of assay in clinical samples was critical. We have performed such validation in archived tissue samples and compared the results with other previously reported methods. From the results, firstly, both fluorescence real-time PCR assays provided higher sensitivity than common PCR. Secondly, the comparison was further performed between two fluorescence real-time PCR assays. All the positive samples detected by Wang’s method were interpreted as positive by our method. However, our method reported additional positive samples (8/110). Since the real-world tissue sample may lead to worse detection performance compared to plasmid-based standard sample, more false-positivity or false-negativity results may be obtained. As a primary screening method, we believed the higher detection rate would be beneficial for the detection of pooling samples containing target with low copies. For the potential false-positivity issue, the sample may be verified with other methods, such as repeated sampling or test, higher template concentration etc.

## Conclusion

A real-time fluorescence PCR assay has been developed for the detection of PCV3, presenting high sensitivity (10 copies/μL) and good specificity (without cross reaction with other porcine viruses). In addition, the performance of our methods in the detection of 110 clinical samples was better than that of with other previously reported methods, presenting good agreement with even higher detection rate. We believed this assay could be widely applied for detecting pooling samples with low copies of PCV3 virus, which may be significant in PCV3 control and prevention.

## Methods

### The reagents and instruments

The common PCR was performed with commercial PCR kit (Promega, USA). The fluorescence PCR was performed with GoTaq® Probe qPCR Master Mix, 1000 rxn (A6102, Promega, USA) and monitored with the 7500 fluorescence PCR instrument (Applied Biosystems, Thermo Fisher Scientific, USA).

### Plasmid construction

The nucleotide sequence of PCV3 capsid protein was synthesized based on the reference sequence retrieved from GenBank (PCV3-US/MO2015, KX778720.1). The sequence was linked to T3 vector and transferred into *E. coli DH5α*. The plasmid genome was extracted with Plasmid MiniPrep Kit (Qiagen, German) and quantified with Nanodrop 2000 (Thermo Fisher Scientific, USA). The copy number of purified capsid was measured and calculated according to the following formula: y copies / μL = [x (ng/μL) × 10^− 9^/ (DNA length× 660)] × 6.02 × 10^23^. The capsid was diluted in a concentration of 10^7^ copies/μL and stored at − 80 °C until use.

### The real-time PCR assay

Primers and probes: The genome sequences of existing 58 PCV3 isolates were retrieved from the GenBank and aligned with Mega software version 6.0. The conserved region of PCV3 sequence was identified as the sequence of capsid, which was selected for designing the primers and probe. The candidate primers and probes were designed with the Primer Premier 5.0 and then synthesized (Invitrogen, Shanghai, China).

Reaction system: a total volume of 20 μL, including 10 μL of GoTaq® Probe qPCR Master Mix (2X); 1.2 μL of primers pair; 0.6 μL of probe; 2 μL of template, 6.2 μL of dH_2_O. The amplification procedure was as follows: 95 °C for 2 min; 40 cycles of 95 °C for 15 s and 60 °C for 1 min.

### Characterization

Sensitivity: The constructed capsid plasmid (with initial concentration of 10^7^ copies/μL) was diluted to 10^6^,10^5^,10^4^,10^3^, 10^2^ and 10^1^ copies/μl. The diluted plasmid was applied for sensitivity assay. The sensitivity test was repeated in triplicate.

Specificity: Common swine viruses and bacterium were tested for specificity, including porcine circovirus type 2 (PCV2, commercial vaccine), pseudorabies virus (PRV, bartha K61, GenBank: JF797217), porcine reproductive and respiratory syndrome virus European strain (PRRSV-EU, Lelystad virus strain, GenBank: M96262) and American strain (PRRSV-NA, JXA1 strain, GenBank: EF112445), classical swine fever (CSFV, C strain, GenBank: Z46258), porcine parvovirus (PPV, NADL-2, ATCC VR-742, GenBank: KF913351.1), *Escherichia coli (Migula) Castellani and Chalmers* (*E.coli.*, ATCC@25922™); *Actinobacillus pleuropneumoniae (Shope) Pohl* et al. (*App.*, ATCC@27088™). The negative control was SPF negative porcine serum obtained from VRMD. The positive control was PCV3 capsid plasmid. All these viruses were stored, cultured and identified by the National Veterinary Diagnosis Center of Chinese Animal Disease Control Center. The specificity test was repeated in triplicate.

Combining the results of sensitivity and specificity, the sequence of primers pair and probe after optimization was provided (Table 1, marked as “This study”).

### Clinical samples

One hundred ten tissue samples were collected from diseased pigs in domestic swine farms, because of dermatitis, respiratory and reproductive disorders. These samples were archived in our institution at − 40 °C for less than 1 year. The whole DNA of the tissue homogenate was extracted with 5*MAGMAX-96 Viral 1 Kit (AM1836–5, ABI, USA) for following assays. The collection of samples involved in our study complied with national guidelines. The study proposal was reviewed and approved from Institutional Review Board of Veterinary Diagnosis Centre, China Animal Disease Control Center. The Written Informed Consent was obtained from the owners of all the involved farms before the samples were applied.

### Detection and comparison with other methods

The DNA of clinical samples after extraction was detected with both the established method in this study and two previously reported methods: the common PCR (Palinski, 2016) and real-time fluorescence PCR methods (Wang, 2017). The common PCR was performed according to the reports of Palinski and determined with 2% agarose gel electrophoresis. The sample with amplification stripe was considered positive. Both real-time fluorescence PCR methods were performed with GoTaq® Probe qPCR Master Mix, 1000rxn (A6102, Promega, USA) and monitored with the 7500 fluorescence PCR instrument. The sample presenting typical amplification curve was interpreted as positive.

## Supplementary information

**Additional file 1. **Supporting information for “Optimized Real-time Fluorescence PCR Assay for the Detection of Porcine Circovirus Type 3 (PCV3)”. **Figure S1.** The repeatability test of sensitivity. **Table S1.** The Ct value of repeatability test for sensitivity).

## Data Availability

The material analyzed during the current study is available from the corresponding author on reasonable request.

## Abbreviations

PCV3: Porcine circovirus type 3
qPCR: quantitative polymerase chain reaction
Ct: Cycle threshold
PCV2: Porcine circovirus type 2
PRV: Pseudorabies virus
PRRSV: Porcine reproductive and respiratory syndrome virus
CSFV: Classical swine fever
PPV: Porcine parvovirus
