# Supplementary material for: Optimized real-time fluorescence PCR assay for the detection of porcine Circovirus type 3 (PCV3)
Source: BMC Vet Res. 2020 Jul 17;16:249. doi: 10.1186/s12917-020-02435-y (PMC7368764; doi:10.1186/s12917-020-02435-y)
Supplement: Supplementary file 1 — Additional file 1. Supporting information for “Optimized Real-time Fluorescence PCR Assay for the Detection of Porcine Circovirus Type 3 (PCV3)”. Figure S1. The repeatability test of sensitivity. Table S1. The Ct value of repeatability test for sensitivity). [file 12917_2020_2435_MOESM1_ESM.docx]

**Supporting information for**

**“Optimized Real-time Fluorescence PCR Assay for the Detection of Porcine Circovirus Type 3 (PCV3)”**

**
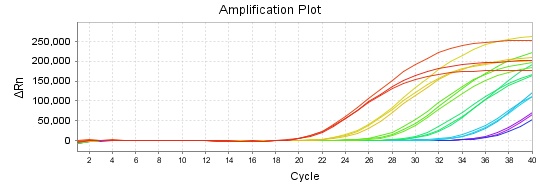
Figure S1.** The repeatability test of sensitivity.

**Table S1. The Ct value of repeatability test for sensitivity**

| Concentration (copies/μL) | 1 | 2 | 3 | Mean | ±Standard deviation |
| --- | --- | --- | --- | --- | --- |
| 10^6^ | 19.28 | 19.34 | 19.26 | 19.3 | ± 0.0 |
| 10^5^ | 23.24 | 23.16 | 23.34 | 23.2 | ± 0.1 |
| 10^4^ | 26.75 | 27.11 | 27.06 | 27.0 | ± 0.2 |
| 10^3^ | 29.48 | 30.19 | 29.67 | 29.8 | ± 0.4 |
| 10^2^ | 33.12 | 33.65 | 33.11 | 33.3 | ± 0.3 |
| 10 | 36.93 | 36.94 | 35.27 | 36.4 | ± 1.0 |
